# Brain MRI analysis for Alzheimer’s disease diagnosis using an ensemble system of deep convolutional neural networks

**DOI:** 10.1186/s40708-018-0080-3

**Published:** 2018-05-31

**Authors:** Jyoti Islam, Yanqing Zhang

**Affiliations:** Department of Computer Science, Georgia State University, Atlanta, GA 30302-5060 USA

**Keywords:** Neurological disorder, Alzheimer’s disease, Deep learning, Convolutional neural network, MRI, Brain imaging

## Abstract

Alzheimer’s disease is an incurable, progressive neurological brain disorder. Earlier detection of Alzheimer’s disease can help with proper treatment and prevent brain tissue damage. Several statistical and machine learning models have been exploited by researchers for Alzheimer’s disease diagnosis. Analyzing magnetic resonance imaging (MRI) is a common practice for Alzheimer’s disease diagnosis in clinical research. Detection of Alzheimer’s disease is exacting due to the similarity in Alzheimer’s disease MRI data and standard healthy MRI data of older people. Recently, advanced deep learning techniques have successfully demonstrated human-level performance in numerous fields including medical image analysis. We propose a deep convolutional neural network for Alzheimer’s disease diagnosis using brain MRI data analysis. While most of the existing approaches perform binary classification, our model can identify different stages of Alzheimer’s disease and obtains superior performance for early-stage diagnosis. We conducted ample experiments to demonstrate that our proposed model outperformed comparative baselines on the Open Access Series of Imaging Studies dataset.

## Background

Alzheimer’s disease (AD) is the most prevailing type of dementia. The prevalence of AD is estimated to be around 5% after 65 years old and is staggering 30% for more than 85 years old in developed countries. It is estimated that by 2050, around 0.64 Billion people will be diagnosed with AD [1]. Alzheimer’s disease destroys brain cells causing people to lose their memory, mental functions and ability to continue daily activities. Initially, Alzheimer’s disease affects the part of the brain that controls language and memory. As a result, AD patients suffer from memory loss, confusion and difficulty in speaking, reading or writing. They often forget about their life and may not recognize their family members. They struggle to perform daily activities such as brushing hair or combing tooth. All these make AD patients anxious or aggressive or to wander away from home. Ultimately, AD destroys the part of the brain controlling breathing and heart functionality which lead to death.

**Fig. 1:**
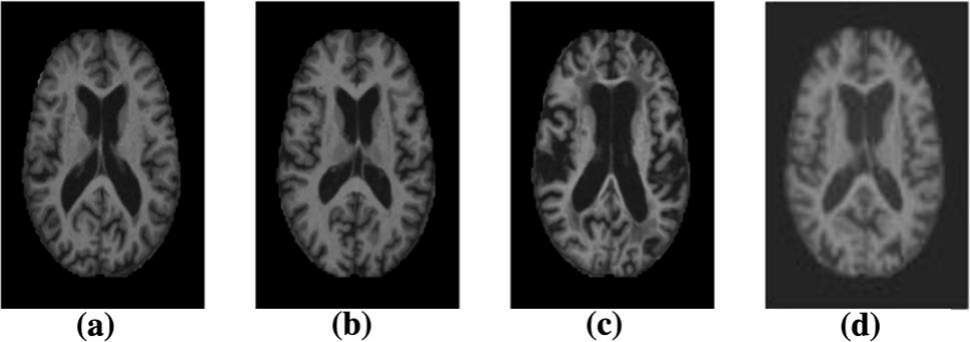
Example of different brain MRI images presenting different Alzheimer’s disease stages. **a** Non-demented; **b** Very mild dementia; **c** Mild dementia; **d** Moderate dementia

There are three major stages in Alzheimer’s disease—very mild, mild and moderate. Detection of Alzheimer’s disease (AD) is still not accurate until a patient reaches moderate AD stage. For proper medical assessment of AD, several things are needed such as physical and neurobiological examinations, Mini-Mental State Examination (MMSE) and patient’s detailed history. Recently, physicians are using brain MRI for Alzheimer’s disease diagnosis. AD shrinks the hippocampus and cerebral cortex of the brain and enlarges the ventricles [2]. Hippocampus is the responsible part of the brain for episodic and spatial memory. It also works as a relay structure between our body and brain. The reduction in hippocampus causes cell loss and damage specifically to synapses and neuron ends. So neurons cannot communicate anymore via synapses. As a result, brain regions related to remembering (short-term memory), thinking, planning and judgment are affected [2]. The degenerated brain cells have low intensity in MRI images [3]. Figure 1 shows some brain MRI images with four different AD stages.

For accurate disease diagnosis, researchers have developed several computer-aided diagnostic systems. They developed rule-based expert systems from 1970s to 1990s and supervised models from 1990s [4]. Feature vectors are extracted from medical image data to train supervised systems. Extracting those features needs human experts that often require a lot of time, money and effort. With the advancement of deep learning models, now we can extract features directly from the images without the engagement of human experts. So researchers are focusing on developing deep learning models for accurate disease diagnosis. Deep learning technologies have achieved major triumph for different medical image analysis tasks such as MRI, microscopy, CT, ultrasound, X-ray and mammography. Deep models showed prominent results for organ and substructure segmentation, several disease detection and classification in areas of pathology, brain, lung, abdomen, cardiac, breast, bone, retina, etc. [4].

As the disease progresses, abnormal proteins (amyloid-$\beta$ [A$\beta$] and hyperphosphorylated tau) are accumulated in the brain of an AD patient. This abnormal protein accumulation leads to progressive synaptic, neuronal and axonal damage. The changes in the brain due to AD have a stereotypical pattern of early medial temporal lobe (entorhinal cortex and hippocampus) involvement, followed by progressive neocortical damage [5]. Such changes occur years before the AD symptoms appear. It looks like the toxic effects of hyperphosphorylated tau and/or amyloid-$\beta$ [A$\beta$] which gradually erodes the brain, and when a clinical threshold is surpassed, amnestic symptoms start to develop. Structural MRI (sMRI) can be used for measuring these progressive changes in the brain due to the AD. Our research work focuses on analyzing sMRI data using deep learning model for Alzheimer’s disease diagnosis.

Machine learning studies using neuroimaging data for developing diagnostic tools helped a lot for automated brain MRI segmentation and classification. Most of them use handcrafted feature generation and extraction from the MRI data. These handcrafted features are fed into machine learning models such as support vector machine and logistic regression model for further analysis. Human experts play a crucial role in these complex multi-step architectures. Moreover, neuroimaging studies often have a dataset with limited samples. While image classification datasets used for object detection and classification have millions of images (for example, ImageNet database [6]), neuroimaging datasets usually contain a few hundred images. But a large dataset is vital to develop robust neural networks. Because of the scarcity of large image database, it is important to develop models that can learn useful features from the small dataset. Moreover, the state-of-the-art deep learning models are optimized to work with natural (every day) images. These models also require a lot of balanced training data to prevent overfitting in the network. We developed a deep convolutional neural network that learned features directly from the input sMRI and eliminated the need for the handcrafted feature generation. We trained our model using the OASIS database [7] that has only 416 sMRI data. Our proposed model can classify different stages of Alzheimer’s disease and outperforms the off-the-shelf deep learning models. Hence, our primary contributions are threefold:

- We propose a deep convolutional neural network that can identify Alzheimer’s disease and classify the current disease stage.
- Our proposed network learns from a small dataset and still demonstrates superior performance for AD diagnosis.
- We present an efficient approach to training a deep learning model with an imbalanced dataset.

The rest of the paper is organized as follows. Section 2 discusses briefly about the related work on AD diagnosis. Section 3 presents the proposed model. Section 4 reports the experimental details and the results. Finally, in Sect. 5, we conclude the paper with our future research direction.

## Related work

Detection of physical changes in brain complements clinical assessments and has an increasingly important role for early detection of AD. Researchers have been devoting their efforts to neuroimaging techniques to measure pathological brain changes related to Alzheimer’s disease. Machine learning techniques have been developed to build classifiers using imaging data and clinical measures for AD diagnosis [8–17]. These studies have identified the significant structural differences in the regions such as the hippocampus and entorhinal cortex between the healthy brain and brain with AD. Changes in cerebrospinal tissues can explain the variations in the behavior of the AD patients [18, 19]. Besides, there is a significant connection between the changes in brain tissues connectivity and behavior of AD patient [20]. The changes causing AD due to the degeneration of brain cells are noticeable on images from different imaging modalities, e.g., structural and functional magnetic resonance imaging (sMRI, fMRI), position emission tomography (PET), single photon emission computed tomography (SPECT) and diffusion tensor imaging (DTI) scans. Several researchers have used these neuroimaging techniques for AD Diagnosis. For example, sMRI [21–26], fMRI [27, 28], PET [29, 30], SPECT [31–33] and DTI [34, 35] have been used for diagnosis or prognosis of AD. Moreover, information from multiple modalities has been combined to improve the diagnosis performance [36–47].

A classic magnetic resonance imaging (MRI)-based automated AD diagnostic system has mainly two building blocks—feature/biomarker extraction from the MRI data and classifier based on those features/biomarkers. Though various types of feature extraction techniques exist, there are three major categories—(1) voxel-based approach, (2) region of interest (ROI)-based approach, and (3) patch-based approach. Voxel-based approaches are independent of any hypothesis on brain structures [48–51]. For example, voxel-based morphometry measures local tissue (i.e., white matter, gray matter and cerebrospinal fluid) density of the brain. Voxel-based approaches exploit the voxel intensities as the classification feature. The interpretation of the results is simple and intuitive in voxel-based representations, but they suffer from the overfitting problem since there are limited (e.g., tens or hundreds) subjects with very high (millions)-dimensional features [52], which is a major challenge for AD diagnosis based on neuroimaging. To achieve more compact and useful features, dimensionality reduction is essential. Moreover, voxel-based approaches suffer from the ignorance of regional information.

Region of interest (ROI)-based approach utilizes the structurally or functionally predefined brain regions and extracts representative features from each region [21, 25, 28, 30, 53–55]. These studies are based on specific hypothesis on abnormal regions of the brain. For example, some studies have adopted gray matter volume [56], hippocampal volume [57–59] and cortical thickness [21, 60]. ROI-based approaches are widely used due to relatively low feature dimensionality and whole brain coverage. But in ROI-based approaches, the extracted features are coarse as they cannot represent small or subtle changes related to brain diseases. The structural or functional changes that occur in the brain due to neurological disorder are typically spread to multiple regions of the brain. As the abnormal areas can be part of a single ROI or can span over multiple ROIs, voxel-based or ROI-based approaches may not efficiently capture the disease-related pathologies. Besides, the region of interest (ROI) definition requires expert human knowledge. Patch-based approaches [23, 61–66] divide the whole brain image into small-sized patches and extract feature vector from those patches. Patch extraction does not require ROI identification, so the necessity of human expert involvement is reduced compared to ROI-based approaches. Compared to voxel-based approaches, patch-based methods can capture the subtle brain changes with significantly reduced dimensionality. Patch-based approaches learn from the whole brain and better captures the disease-related pathologies that results in superior diagnosis performance. However, there is still challenges to select informative patches from the MRI images and generate discriminative features from those patches.

A large number of research works focused on developing advanced machine learning models for AD diagnosis using MRI data. Support vector machine SVM), logistic regressors (e.g., Lasso and Elastic Net), sparse representation-based classification (SRC), random forest classifier, etc., are some widely used approaches. For example, Kloppel et al. [50] used linear SVM to detect AD patients using T1 weighted MRI scan. Dimensional reduction and variations methods were used by Aversen [67] to analyze structural MRI data. They have used both SVM binary classifier and multi-class classifier to detect AD from MRI images. Vemuri et al. [68] used SVM to develop three separate classifiers with MRI, demographic and genotype data to classify AD and healthy patients. Gray [69] developed a multimodal classification model using random forest classifier for AD diagnosis from MRI and PET data. Er et al. [70] used gray-level co-occurrence matrix (GLCM) method for AD classification. Morra et al. [71] compared several model’s performances for AD detection including hierarchical AdaBoost, SVM with manual feature and SVM with automated feature. For developing these classifiers, typically predefined features are extracted from the MRI data. However, training a classifier independent from the feature extraction process may result in sub-optimal performance due to the possible heterogeneous nature of the classifier and features [72].

Recently, deep learning models have been famous for their ability to learn feature representations from the input data. Deep learning networks use a layered, hierarchical structure to learn increasingly abstract feature representations from the data. Deep learning architectures learn simple, low-level features from the data and build complex high-level features in a hierarchy fashion. Deep learning technologies have demonstrated revolutionary performance in several areas, e.g., visual object recognition, human action recognition, natural language processing, object tracking, image restoration, denoising, segmentation tasks, audio classification and brain–computer interaction. In recent years, deep learning models specially convolutional neural network (CNN) have demonstrated excellent performance in the field of medical imaging, i.e., segmentation, detection, registration and classification [4]. For neuroimaging data, deep learning models can discover the latent or hidden representation and efficiently capture the disease-related pathologies. So, recently researchers have started using deep learning models for AD and other brain disease diagnosis.

Gupta et al. [62] have developed a sparse autoencoder model for AD, mild cognitive impairment (MCI) and healthy control (HC) classification. Payan and Montana [65] trained sparse autoencoders and 3D CNN model for AD diagnosis. They also developed a 2D CNN model that demonstrated nearly identical performance. Brosch et al. [73] developed a deep belief network model and used manifold learning for AD detection from MRI images. Hosseini-Asl et al. [74] adapted a 3D CNN model for AD diagnostics. Liu and Shen [75] developed a deep learning model using both unsupervised and supervised techniques and classified AD and MCI patients. Liu et al. [76] have developed a multimodal stacked autoencoder network using zero-masking strategy. Their target was to prevent loss of any information of the image data. They have used SVM to classify the neuroimaging features obtained from MR/PET data. Sarraf and Tofighi [77] used fMRI data and deep LeNet model for AD detection. Suk et al. [23, 42, 78, 79] developed an autoencoder network-based model for AD diagnosis and used several complex SVM kernels for classification. They have extracted low- to mid-level features from magnetic current imaging (MCI), MCI-converter structural MRI, and PET data and performed classification using multi-kernel SVM. Cárdenas-Peña et al. [80] have developed a deep learning model using central kernel alignment and compared the supervised pre-training approach to two unsupervised initialization methods, autoencoders and principal component analysis (PCA). Their experiment shows that SAE with PCA outperforms three hidden layers SAE and achieves an increase of 16.2% in overall classification accuracy.

So far, AD is detected at a much later stage when treatment can only slow the progression of cognitive decline. No treatment can stop or reverse the progression of AD. So, early diagnosis of AD is essential for preventive and disease-modifying therapies. Most of the existing research work on AD diagnosis focused on binary classification problems, i.e., differentiating AD patients from healthy older adults. However, for early diagnosis, we need to distinguish among current AD stages, which makes it a multi-class classification problem. In our previous work [81], we developed a very deep convolutional network and classified the four different stages of the AD—non-demented, very mild dementia, mild dementia and moderate dementia. For our current work, we improved the previous model [81], developed an ensemble of deep convolutional neural networks and demonstrated better performance on the Open Access Series of Imaging Studies (OASIS) dataset [7].

## Methods

### Formalization

Let $$ x = \left\{ x_{i}, \, i = 1,\ldots , N \right\} $$, a set of MRI data with $$x_{i} \in [0, 1, 2, \ldots , L-1]^{^{h*w*l}}$$, a three-dimensional (3D) image with *L* grayscale values, $$h*w*l$$ voxels and $$y \in \left\{ {0, 1, 2, 3}\right\} $$, one of the stages of AD where 0, 1, 2 and 3 refer to non-demented, very mild dementia, mild dementia and moderate dementia, respectively. We will construct a classifier,

1 $$ f: X \rightarrow Y ; \, x \mapsto y, $$

which predicts a label y in response to an input image x with minimum error rate. Mainly, we want to determine this classifier function *f* by an optimal set of parameters $w \in {\mathbb {R}}^{P}$ (where *P* can easily be in the tens of millions), which will minimize the loss or error rate of prediction. The training process of the classifier would be an iterative process to find the set of parameters *w*, which minimizes the classifier’s loss

2 $$ L(w, X) = \frac{1}{n}\sum _{i=1}^{n}l (f(x_{i}, w), \widehat{c_i}) $$

where $x_i$ is *i*th image of *X*, $f(x_{i}, w)$ is the classifier function that predicts the class $c_i$ of $x_i$ given *w*, $\widehat{c_{i}}$ is the ground-truth class for *i*th image $x_{i}$ and $l(c_i,\widehat{c_{i}})$ is the penalty function for predicting $c_i$ instead of $\widehat{c_{i}}$ . We set *l* to the loss of cross-entropy,

3 $$ l = -\sum _{i}{\widehat{c_{i}}}\, \log \, c_i $$

### Data selection

In this study, we use the OASIS dataset [7] prepared by Dr. Randy Buckner from the Howard Hughes Medical Institute (HHMI) at Harvard University, the Neuroinformatics Research Group (NRG) at Washington University School of Medicine, and the Biomedical Informatics Research Network (BIRN). There are 416 subjects aged 18–96, and for each of them, 3 or 4 T1-weighted sMRI scans are available. Hundred of the patients having age over 60 are included in the dataset with very mild to moderate AD.

### Data augmentation

Data augmentation refers to artificially enlarging the dataset using class-preserving perturbations of individual data to reduce the overfitting in neural network training [82]. The reproducible perturbations will enable new sample generation without changing the semantic meaning of the image. Since manually sourcing of additional labeled image is difficult in medical domain due to limited expert knowledge availability, data augmentation is a reliable way to increase the size of the dataset. For our work, we developed an augmentation scheme involving cropping for each image. We set the dimension of the crop similar to the dimension of the proposed deep CNN classifier. Then, we extracted three crops from each image, each for one of the image plane: axial or horizontal plane, coronal or frontal plane, and sagittal or median plane. For our work, we use 80% data from the OASIS dataset as training set and 20% as test dataset. From the training dataset, a random selection of 10% images is used as validation dataset. The augmentation process is performed separately for the train, validation and test dataset. One important thing to consider is the data augmentation process is different from classic cross-validation scheme. Data augmentation is used to reduce overfitting in a vast neural network while training with a small dataset. On the other hand, cross-validation is used to derive a more accurate estimate of model prediction performance. Cross-validation technique is computationally expensive for a deep convolutional neural network training as it takes an extensive amount of time.

### Network architecture

Our proposed network is an ensemble of three deep convolutional neural networks with slightly different configurations. We made a considerable amount of effort for the design of the proposed system and the choice of the architecture. All the individual models have a common architectural pattern consisted of four basic operations:

- convolution
- batch normalization [83]
- rectified linear unit, and
- pooling

**Fig. 2:**
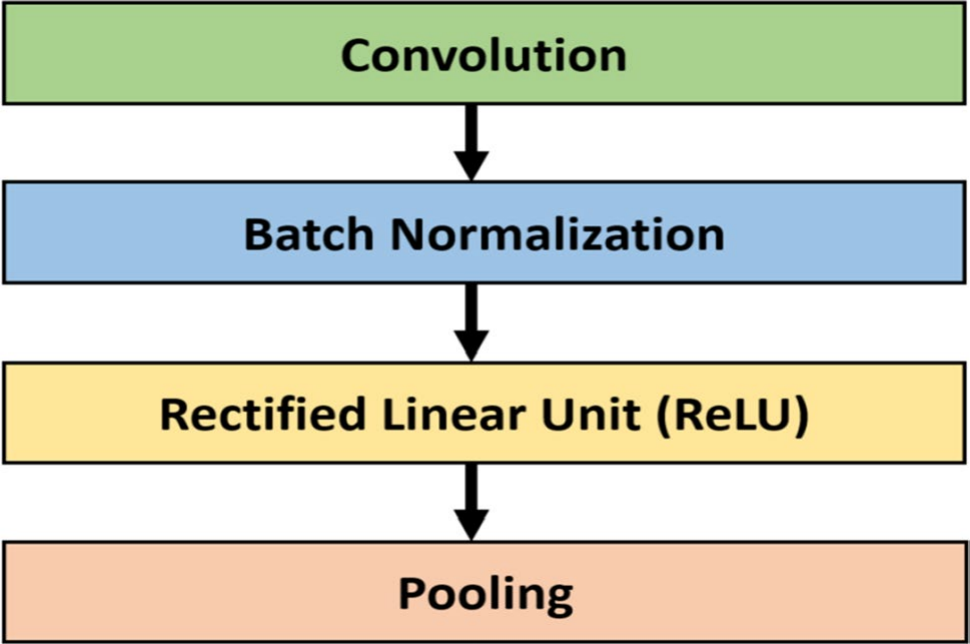
Common building block of the proposed ensemble model

**Fig. 3:**
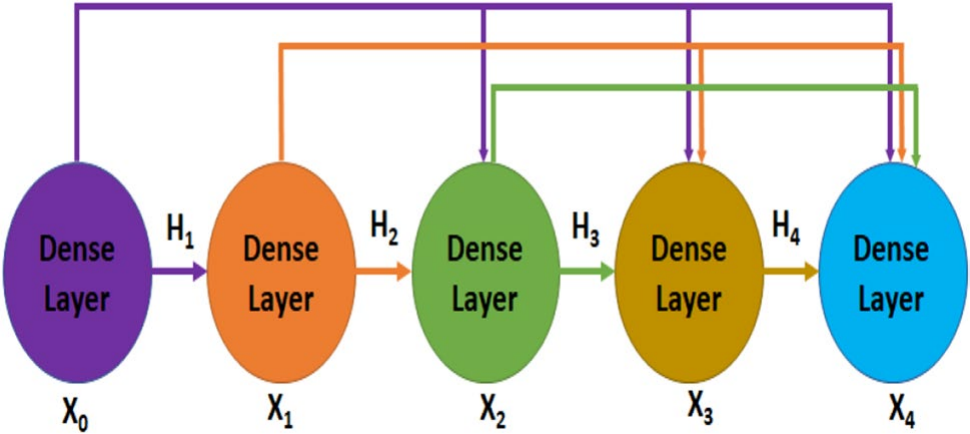
Illustration of dense connectivity with a 5-layer dense block

**Fig. 4:**
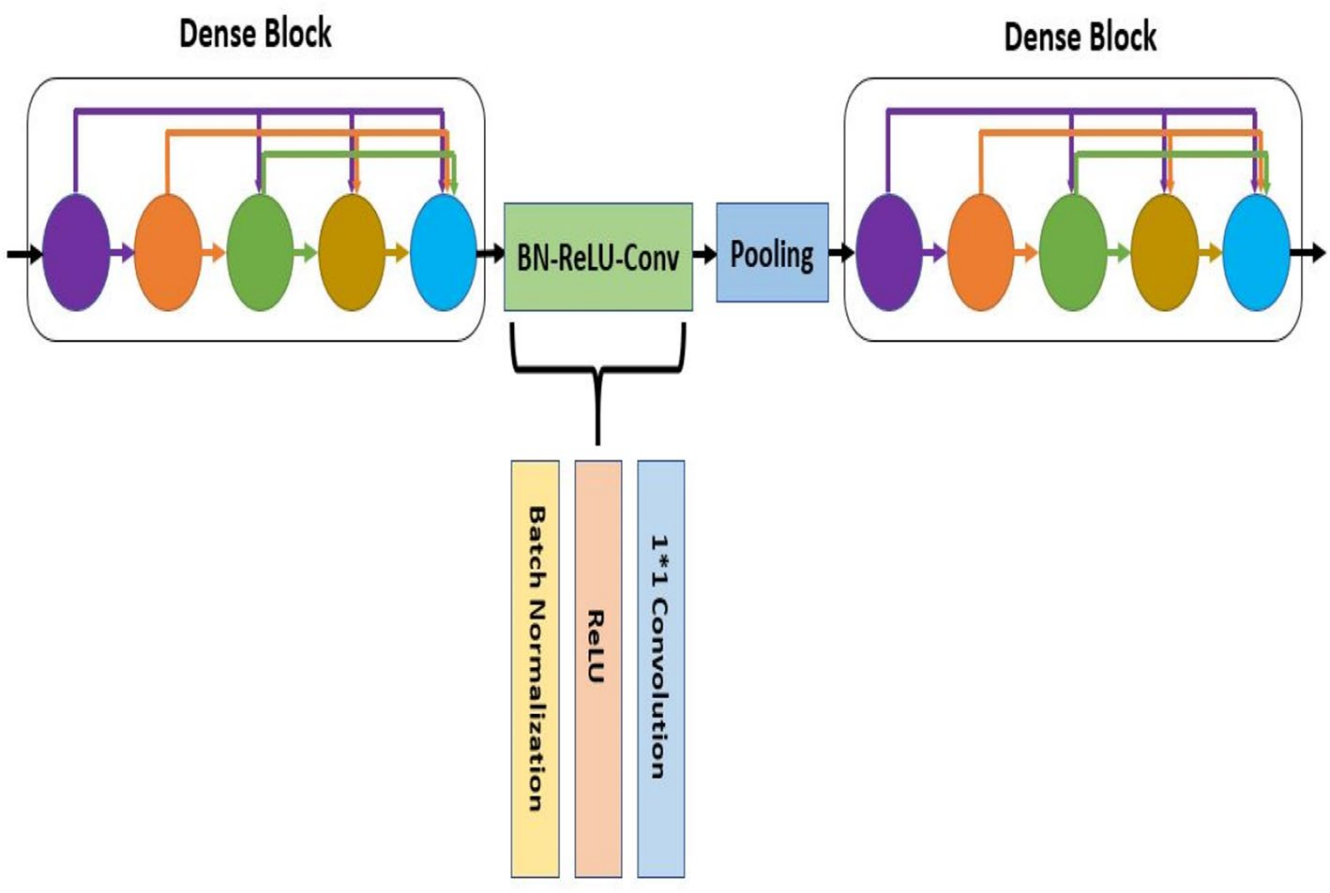
Illustration of two dense blocks and their intermediate connection

Each of the individual convolutional neural networks has several layers performing these four basic operations illustrated in Fig. 2. The layers in the model follow a particular connection pattern known as dense connectivity [84] as shown in Fig. 3. The dense connections have a regularizing effect that reduces overfitting in the network while training with a small dataset. We keep these layers very narrow (e.g., 12 filters per layer) and connect each layer to every other layer. Similar to [84], we will refer to the layers as dense layer and combination of the layers as dense block. Since all the dense layers are connected to each other, the *i*th layer receives the feature maps ($h_{0}, h_{1}, h_{2}, \ldots , h_{i-1}$), from all previous layers ($0, 1, 2, \ldots , i-1)$. Consequently, the network has a global feature map set, where each layer adds a small set of feature maps. In times of training, each layer can access the gradients from the loss function as well as the original input. Therefore, the flow of information improves, and gradient flow becomes stronger in the network. Figure 4 shows the intermediate connection between two dense blocks.

**Fig. 5:**
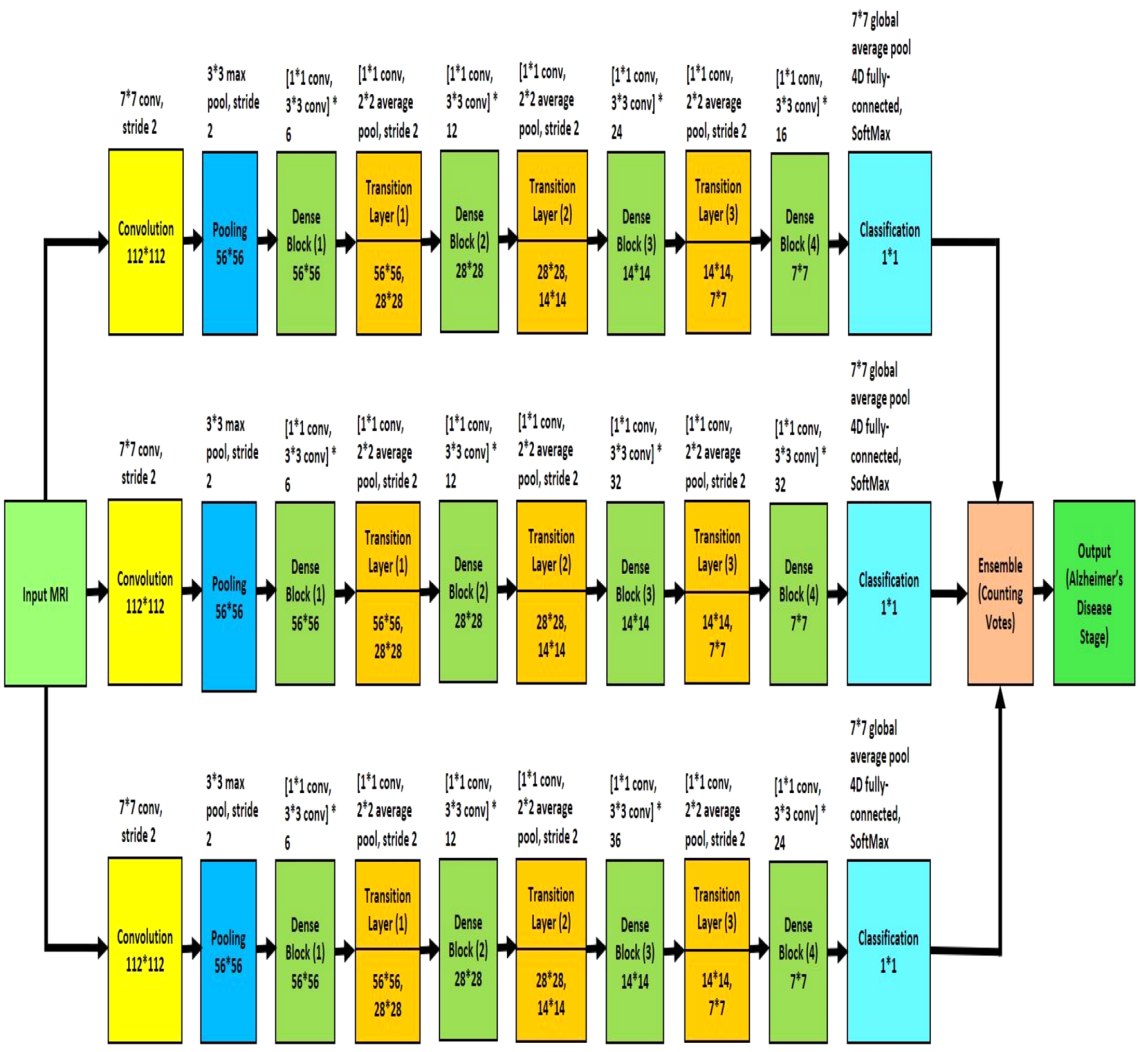
Block diagram of proposed Alzheimer’s disease diagnosis framework

For the design of the proposed system, we experimented with several different deep learning architectures and finally developed an ensemble of three homogeneous deep convolution neural networks. The proposed model is shown in Fig. 5. We will refer to the individual models as $\hbox {M}_{1}$, $\hbox {M}_{2}$ and $\hbox {M}_{3}$. In Fig. 5, the top network is $\hbox {M}_{1}$, the middle network is $\hbox {M}_{2}$, and the bottom network is $\hbox {M}_{3}$. Each of the models consists of several convolution layers, pooling layers, dense blocks and transition layers. The transition layer is a combination of batch normalization layer, a 1*1 convolutional layer followed by a 2 * 2 average pooling layer with stride 2. Batch normalization [83] acts as a regularizer and speeds up the training process dramatically. Traditional normalization process (shifting inputs to zero-mean and unit variance) is used as a preprocessing step. Normalization is applied to make the data comparable across features. When the data flow inside the network at the time of training process, the weights and parameters are continuously adjusted. Sometimes these adjustments make the data too big or too small, a problem referred as ‘Internal Covariance Shift.’ Batch normalization largely eliminates this problem. Instead of doing the normalization at the beginning, batch normalization is performed to each mini-batches along with SGD training. If ${\mathfrak {B}} = \left\{ {x_1, x_2,\ldots ,x_m} \right\}$ is a mini-batch of *m* activations value, the normalized values are $(\widehat{x}_1, \widehat{x}_2,\ldots ,\widehat{x}_m)$ and the linear transformations are ${y_1, y_2,\ldots ,y_m}$, then batch normalization is referred to the transform:

4 $$ BN_{\gamma , \beta }: {x_1, x_2,\ldots ,x_m} \rightarrow {y_1, y_2,\ldots ,y_m} $$

Considering $\gamma , \beta$ the parameters to be learned and $\epsilon$, a constant added to the mini-batch variance for numerical stability, batch normalization is given by the following equations:

5a $$ \mu _{\mathfrak {B}} \leftarrow \frac{1}{m}\sum _{i=1}^{m} x_i $$

5b $$ \sigma ^2_{\mathfrak {B}} \leftarrow \frac{1}{m}\sum _{i=1}^{m} (x_i - \mu _{\mathfrak {B}})^2 $$

5c $$ \widehat{x}_i \leftarrow \frac{{x}_i - \mu _{\mathfrak {B}}}{\sqrt{\sigma ^2_{\mathfrak {B}} + \epsilon } } $$

5d $$ y_i \leftarrow \gamma \widehat{x_i} + \beta \equiv BN_{\gamma , \beta } (x_i) $$

where $\mu _{\mathfrak {B}}$ is mini-batch mean and $\sigma ^2_{\mathfrak {B}}$ is mini-batch variance [83].

Though each model has four dense blocks, they differ in the number of their internal 1*1 convolution and 3*3 convolution layers. The first model, $\hbox {M}_{1}$, has six (1 * 1 convolution and 3 * 3 convolution layers) in the first dense block, twelve (1*1 convolution and 3*3 convolution layers) in the second dense block, twenty-four (1*1 convolution and 3*3 convolution layers) in the third dense block and sixteen (1*1 convolution and 3*3 convolution layers) in the fourth dense block. The second model, $\hbox {M}_{2}$, and third model, $\hbox {M}_{3}$, have (6, 12, 32, 32) and (6, 12, 36, 24) arrangement respectively. Because of the dense connectivity, each layer has direct connections to all subsequent layers, and they receive the feature maps from all preceding layers. So, the feature maps work as global state of the network, where each layer can add their own feature map. The global state can be accessed from any part of the network and how much each layer can contribute to is decided by the growth rate of the network. Since the feature maps of different layers are concatenated together, the variation in the input of subsequent layers increases and results in more efficiency.

**Fig. 6:**
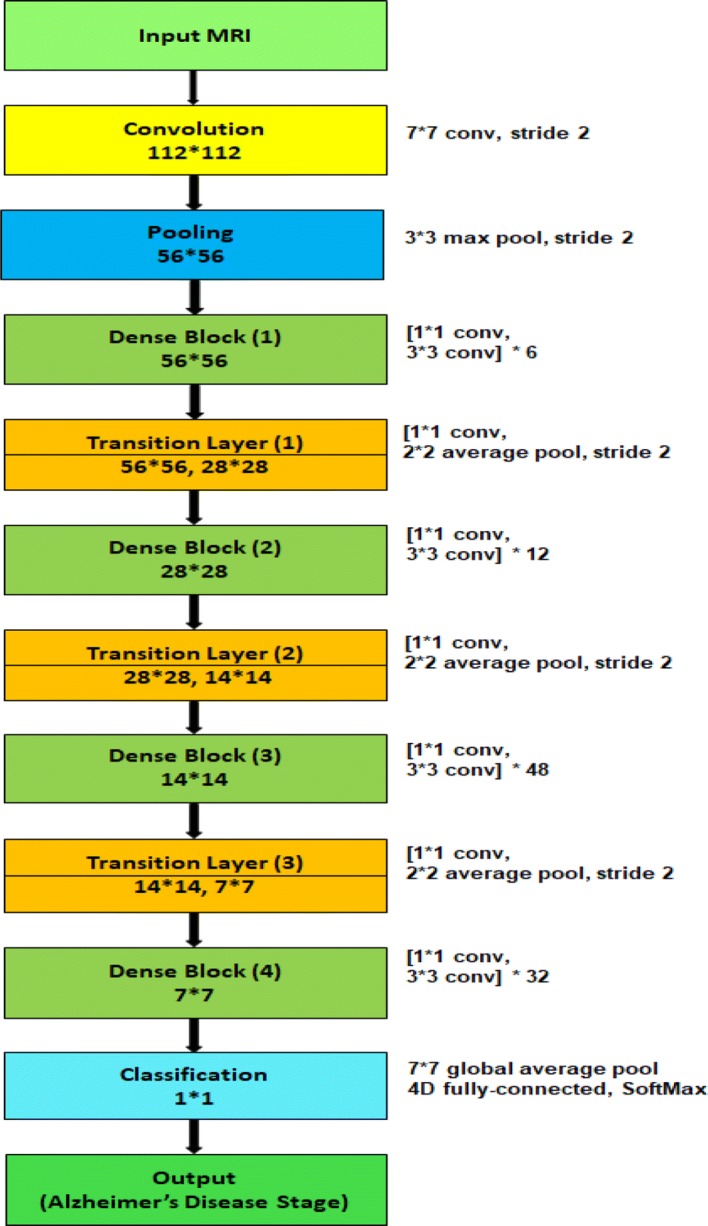
Block diagram of individual model $\hbox {M}_{4}$

The input MRI is 3D data, and our proposed model is a 2D architecture, so we devise an approach to convert the input data to 2D images. For each MRI data, we created patches from three physical planes of imaging: axial or horizontal plane, coronal or frontal plane, and sagittal or median plane. These patches are fed to the proposed network as input. Besides, this data augmentation technique increases the number of samples in training dataset. The size of each patch is 112*112. We trained the individual models separately, and each of them has own softmax layer for classification decision. The softmax layers have four different output classes: non-demented, very mild, mild and moderate AD. The individual models take the input image and generate its learned representation. The input image is classified to any of the four output classes based on this feature representation. To measure the loss of each of these models, we used cross-entropy. The softmax layer takes the learned representation, $f_{i}$, and interprets it to the output class. A probability score, $p_{i}$, is also assigned for the output class. If we define the number of output classes as m, then we get

6 $$ p_{i} = \frac{\exp (f{_i})}{\sum _{i}{} \exp (f{_i})}, i=1,\ldots ,m $$

and

7 $$ L = -\sum _{i}{} t_i \log (p_i) $$

where L is the loss of cross-entropy of the network. Backpropagation is used to calculate the gradients of the network. If the ground truth of an MRI data is denoted as $t_{i}$, then

8 $$ \frac{ \partial L}{ \partial f_i} = p_i - t_i $$

To handle the imbalance in the dataset, we used cost-sensitive training [85]. A cost matrix $\xi$ was used to modify the output of the last layer of the individual networks. Since the less frequent classes (very mild dementia, mild dementia, moderate dementia) are underrepresented in the training dataset, the output of the networks was modified using the cost matrix $\xi$ to give more importance to these classes. If *o* is the output of the individual model, *p* is the desired class and *L* is the loss function, then y denotes the modified output:

9 $$ y^i = L(\xi _p, o^i),:\quad y^i_p \ge y^i_j \quad \forall j\ne p $$

The loss function is modified as:

10 $$ L = -\sum _{n}{} t_n \log (y_n) $$

where $y_n$ incorporates the class-dependent cost $\xi$ and is related to the output $o_n$ via the softmax function [85]:

11 $$ y_n = \frac{\xi _{p,n}\exp (o_n)}{\sum _k \xi _{p,k} \exp (o_k)} $$

The weight of a particular class is dependent on the number of samples of that class. If class *r* has *q* times more samples than those of *s*, the target is to make one sample of class *s* to be as important as *q* samples of class *r*. So, the class weight of *s* would be *q* times more than the class weight of *r*.

We optimized the individual models with the stochastic gradient descent (SGD) algorithm. For regularization, we used early stopping. We split the training dataset into a training set and a cross-validation set in 9:1 proportion. Let $L_{tr}(t)$ and $L_{va}(t)$ are the average error per example over the training set and validation set respectively, measured after *t* epoch. Training was stopped as soon as it reached convergence, i.e., validation error $L_{va}(t)$ does not improve for *t* epoch and $L_{va}(t) > L_{va}(t-1)$. We used Nesterov momentum optimization with Stochastic Gradient Descent (SGD) algorithm for minimizing the loss of the network. Given an objective function $f(\theta )$ to be minimized, classic momentum is given by the following pair of equations:

12a $$ v_t = \mu v_{t-1} - \epsilon \nabla f (\theta _{t-1}) $$

12b $$ \theta _{t} = \theta _{t-1} + v_t $$

where $v_t$ refers to the velocity, $\epsilon >0$ is the learning rate, $\mu \in [0, 1]$ is the momentum coefficient and $\nabla f \theta _{t}$ is the gradient at $\theta _{t}$. On the other hand, Nesterov momentum is given by:

13a $$ v_t = \mu v_{t-1} - \epsilon \nabla f (\theta _{t-1} + \mu v_{t-1}) $$

13b $$ \theta _{t} = \theta _{t-1} + v_t $$

The output classification labels of the three individual model are ensembled together using majority voting technique. Each classifier 'votes' for a particular class, and the class with the majority votes would be assigned as the label for the input MRI data.

## Results and discussion

### Experimental settings

We implemented the proposed model using Tensorflow [86], Keras[87] and Python on a Linux X86-64 machine with AMD A8 CPU, 16 GB RAM and NVIDIA GeForce GTX 770. We applied the SGD training with a mini-batch size of 64, a learning rate of 0.01, a weight decay of 0.06 and a momentum factor of 0.9 with Nesterov optimization. We applied early stopping in the SGD training process, while there was no improvement (change of less than 0.0001) in validation loss for last six epoch.

**Fig. 7:**
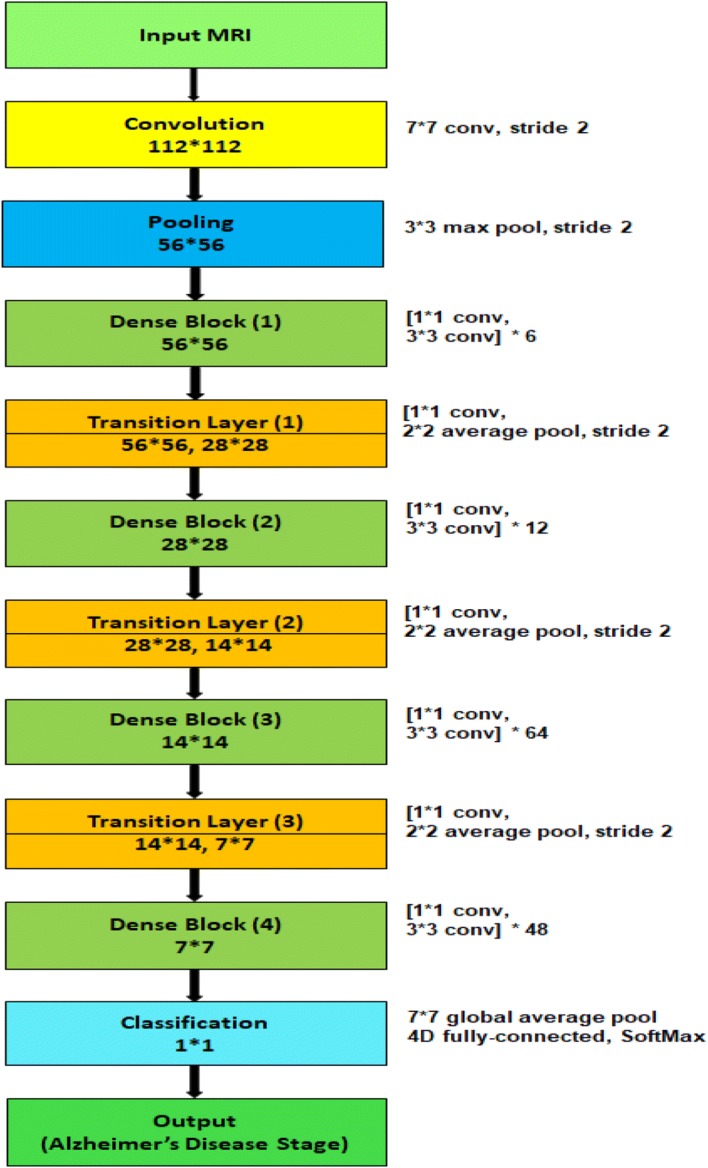
Block diagram of individual model $\hbox {M}_{5}$

To validate the effectiveness of the proposed AD detection and classification model, we developed two baseline deep CNN, Inception-v4 [88] and ResNet [89] and modified their architecture two classify 3D brain MRI data. Besides, we developed two different models, $\hbox {M}_{4}$ and $\hbox {M}_{5}$ having similar architecture like $\hbox {M}_{1}$, $\hbox {M}_{2}$ and $\hbox {M}_{3}$ model except for the number of layers in the dense block. $\hbox {M}_{4}$ has six (1*1 convolution and 3*3 convolution layers) in the first dense block, twelve (1*1 convolution and 3*3 convolution layers) in the second dense block, forty-eight (1*1 convolution and 3*3 convolution layers) in the third dense block and thirty-two (1*1 convolution and 3*3 convolution layers) in the fourth dense block (Fig. 6). The layers in the dense blocks of $\hbox {M}_{5}$ have the arrangement 6, 12, 64, 48 as shown in Fig. 7. Additionally, we implemented two variants of our proposed model using $\hbox {M}_{4}$ and $\hbox {M}_{5}$.

- For the first variant, we implemented an ensemble of four deep convolutional neural networks: $\hbox {M}_{1}$, $\hbox {M}_{2}$, $\hbox {M}_{3}$ and $\hbox {M}_{4}$. We will refer to this model as $\hbox {E}_{1}$.
- For the second variant, we implemented an ensemble system of five deep convolutional neural networks: $\hbox {M}_{1}$, $\hbox {M}_{2}$, $\hbox {M}_{3}$, $\hbox {M}_{4}$ and $\hbox {M}_{5}$. We will refer to this model as $\hbox {E}_{2}$.

### Performance metric

Four metrics are used for quantitative evaluation and comparison, including accuracy, positive predictive value (PPV) or precision, sensitivity or recall, and the harmonic mean of precision and sensitivity (f1-score). We denote TP, TN, FP and FN as true positive, true negative, false positive and false negative, respectively. The evaluation metrics are defined as:

$$\begin{aligned} {\text {accuracy}} &= \frac{({\text {TP}}+{\text {TN}})}{({\text {TP}}+{\text {FP}}+{\text {FN}}+{\text {TN}})}\\ {\text {precision}} &= \frac{{\text {TP}}}{({\text {TP}}+{\text {FP}})}\\ {\text {recall}} &= \frac{{\text {TP}}}{({\text {TP}}+{\text {FN}})}\\ f1{\text {-score}} &= \frac{(2{\text {TP}})}{(2{\text {TP}}+{\text {FP}}+{\text {FN}})} \end{aligned}$$

### Dataset

The OASIS dataset [7] has 416 data samples. The dataset is divided into a training dataset and a test dataset in 4:1 proportion. A validation dataset was prepared using 10% data from the training dataset.

### Results

**Fig. 8:**
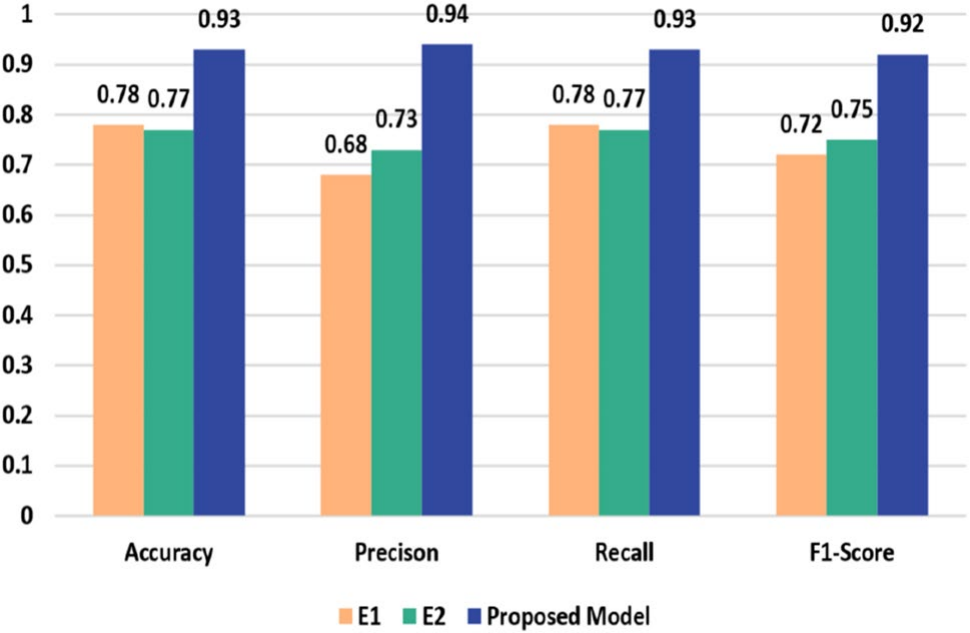
Performance comparison of the proposed model and the variants

We report the classification performance of $\hbox {M}_{1}$, $\hbox {M}_{2}$, $\hbox {M}_{3}$, $\hbox {M}_{4}$ and $\hbox {M}_{5}$ model in Tables 1, 2, 3, 4 and 5, respectively. From the results, we notice that $\hbox {M}_{1}$, $\hbox {M}_{2}$ and $\hbox {M}_{3}$ model are the top performers among all models. So, we choose the ensemble of $\hbox {M}_{1}$, $\hbox {M}_{2}$, $\hbox {M}_{3}$ for our final architecture. Besides, the variants $\hbox {E}_{1}$ ($\hbox {M}_{1}+\hbox {M}_{2}+\hbox {M}_{3}+\hbox {M}_{4}$) and $\hbox {E}_{2}$ ($\hbox {M}_{1}+\hbox {M}_{2}+\hbox {M}_{3}+\hbox {M}_{4}+\hbox {M}_{5}$) demonstrate inferior performance compared to the ensemble of $\hbox {M}_{1}$, $\hbox {M}_{2}$, $\hbox {M}_{3}$ (proposed model) as shown in Fig. 8. From Fig. 8, we notice that $\hbox {E}_{1}$ model has an accuracy of 78% with 68% precision, 78% recall and 72% f1 score. On the other hand, the $\hbox {E}_{2}$ model demonstrates 77% accuracy with 73% precision, 77% recall and 75% f1-score.

**Table 1:** Classification performance of $\hbox {M}_{1}$ model

Class	Precision	Recall	*f*1-score	Support
Non-demented	0.99	0.99	0.99	73
Very mild	0.75	0.50	0.60	6
Mild	0.62	0.71	0.67	7
Moderate	0.33	0.50	0.40	2
Avg/total	0.93	0.92	0.92	88

**Table 2:** Classification performance of $\hbox {M}_{2}$ model

Class	Precision	Recall	*f*1-score	Support
Non-demented	0.88	0.95	0.91	73
Very mild	0.00	0.00	0.00	6
Mild	0.25	0.29	0.27	7
Moderate	0.00	0.00	0.00	2
Avg/total	0.75	0.81	0.78	88

**Table 3:** Classification performance of $\hbox {M}_{3}$ model

Class	Precision	Recall	*f*1-score	Support
Non-demented	0.99	0.96	0.97	73
Very mild	0.50	0.33	0.40	6
Mild	0.45	0.71	0.56	7
Moderate	0.50	0.50	0.50	2
Avg/total	0.90	0.89	0.89	88

**Table 4:** Classification performance of $\hbox {M}_{4}$ model

Class	Precision	Recall	*f*1-score	Support
Non-Demented	0.92	0.67	0.77	73
Very Mild	0.00	0.00	0.00	6
Mild	0.17	0.60	0.26	7
Moderate	0.00	0.00	0.00	2
Avg/Total	0.77	0.61	0.66	88

**Table 5:** Classification performance of $\hbox {M}_{5}$ model

Class	Precision	Recall	*f*1-score	Support
Non-demented	0.80	0.94	0.86	73
Very Mild	0.00	0.00	0.00	6
Mild	0.22	0.14	0.17	7
Moderate	0.00	0.00	0.00	2
Avg/Total	0.64	0.74	0.68	88

**Table 6:** Performance of the proposed ensembled model

Class	Precision	Recall	*f*1-score	Support
Non-demented	0.97	1.00	0.99	73
Very mild	1.00	0.33	0.50	6
Mild	0.67	0.86	0.75	7
Moderate	0.50	0.50	0.50	2
Avg/total	0.94	0.93	0.92	88

**Fig. 9:**
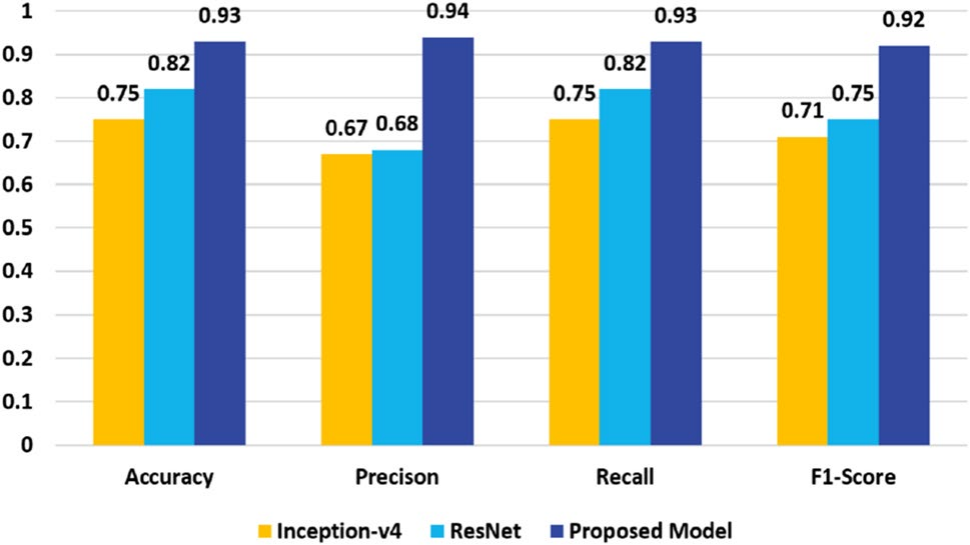
Performance comparison of the proposed model and the baseline deep CNNs

Table 6 shows the per-class classification performance of our proposed ensembled model on the OASIS dataset [7]. The accuracy of the proposed model is 93.18% with 94% precision, 93% recall and 92% f1-score. The performance comparison of classification results of the proposed ensembles model, and the two baseline deep CNN models are presented in Fig. 9. Inception-v4 [88] and ResNet [89] have demonstrated outstanding performance for object detection and classification. The reason behind their poor performance for AD detection and classification can be explained by the lack of enough training dataset.

**Fig. 10:**
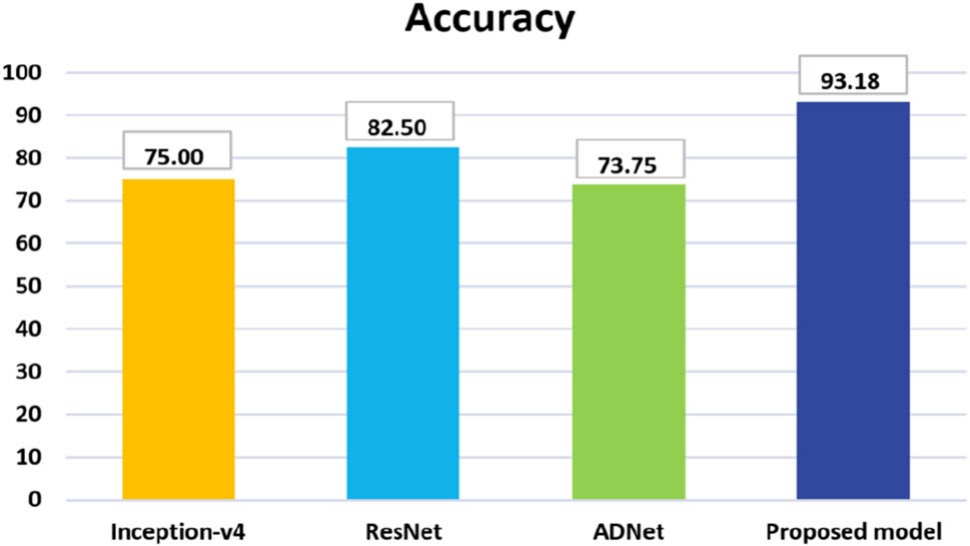
Comparison of accuracy on the OASIS dataset [7]

Since these two networks are very deep neural networks, so without a large dataset, training process would not work correctly. On the other hand, the depth of our model is relatively low, and all the layers are connected to all preceding layers. So, there is a strong gradient flow in times of training that eliminates the ‘Vanishing gradient’ problem. In each training iteration, all the weights of a neural network receive an update proportional to the gradient of the error function concerning the current weight. But in some cases, the gradient will be vanishingly small and consequently prevent the weight from changing its value. It may completely stop the neural network from further training in worst-case scenario. Our proposed model does not suffer this ‘Vanishing gradient’ problem, have better feature propagation and provides better classification result even for the small dataset. The performance comparison of classification results of the proposed ensembled model, the baseline deep CNN models and the most recent work, ADNet [81] is presented in Fig. 10. It can be observed that proposed ensembled model achieves encouraging performance and outperforms the other models.

## Conclusion

We made an efficient approach to AD diagnosis using brain MRI data analysis. While the majority of the existing research works focuses on binary classification, our model provides significant improvement for multi-class classification. Our proposed network can be very beneficial for early-stage AD diagnosis. Though the proposed model has been tested only on AD dataset, we believe it can be used successfully for other classification problems of medical domain. Moreover, the proposed approach has strong potential to be used for applying CNN into other areas with a limited dataset. In future, we plan to evaluate the proposed model for different AD datasets and other brain disease diagnosis.
